# *Pseudomonas aeruginosa* Isolation from Urine Culture in Hospitalised Patients: Incidence of Complicated Urinary Tract Infections and Asymptomatic Bacteriurias and Impact on Treatment of the EUCAST 2020 Update

**DOI:** 10.3390/antibiotics13121206

**Published:** 2024-12-11

**Authors:** Carlo Pallotto, Paolo Milani, Caterina Catalpi, Donatella Pietrella, Giuseppe Curcio, Filippo Allegrucci, Anna Gidari, Elisabetta Svizzeretto, Giovanni Genga, Andrea Tommasi, Antonella Mencacci, Daniela Francisci

**Affiliations:** 1 Infectious Diseases Clinic, Santa Maria della Misericordia Hospital, Department of Medicine, University of Perugia, Piazzale Menghini 1, 06132 Perugia, Italy; 2 Microbiology, Santa Maria della Misericordia Hospital, Piazzale Menghini 1, 06132 Perugia, Italy; 3 Medical Microbiology, Department of Medicine and Surgery, University of Perugia, Piazzale Menghini 1, 06132 Perugia, Italy; 4 Infectious Diseases Clinic, Santa Maria Hospital, Department of Medicine, University of Perugia, Viale Tristano di Joannuccio, 05100 Terni, Italy

**Keywords:** *Pseudomonas aeruginosa*, urinary tract infection, asymptomatic bacteriuria, EUCAST 2020 update, antimicrobial stewardship, carbapenem, anti-Pseudomonas cephalosporins, cefepime

## Abstract

**Background.** Urinary tract infections (UTIs) and asymptomatic bacteriurias (ABU) represent a large field of interest for antimicrobial stewardship programmes especially after 2020 EUCAST update in antimicrobial susceptibility testing interpretation and the possible related increase in carbapenems’ prescription rate. The aim of this study was to evaluate the impact of the 2020 EUCAST update on antibiotic prescription in UTI due to *Pseudomonas aeruginosa* organism and their characteristics. **Methods.** A retrospective observational study. We enrolled all the patients with *P. aeruginosa* isolation from urine, admitted to our hospital from 2018 to 2021. We compared demographic, clinical, and microbiological characteristics and treatment between cases before 2020 EUCAST update (period A, 2018–2019) and after it (period B, 2020–2021). **Results.** A total of 643 cases was analysed, 278 in period A and 365 in period B; 65% were ABU. Carbapenems’ prescription rate significantly increased in period B when considering ABU alone (21.4% vs. 41%, *p* = 0.016) and all the treated cases (treated ABU and UTI; 27.8% vs. 41.4%, *p* = 0.013); anti-Pseudomonas cephalosporins prescription significantly decreased in period B when considering ABU alone (15.7% vs. 3.6%, *p* = 0.021), UTI alone (20.7% vs. 5.9%, *p* = 0.009) and all the treated cases (18.5% vs. 5.9%, *p* = 0.001). **Conclusions.** The 2020 EUCAST update could have contributed to an increase in carbapenem prescriptions. UTI and ABU represent a large field of interest for stewardship interventions both from a diagnostic and therapeutic point of view.

## 1. Introduction

Urinary tract infections (UTIs) represent the most frequent infections both in the community and in the hospital setting [1]. Moreover, very commonly a positive urine culture could not be associated to an infection but to an asymptomatic bacteriuria (ABU) that should not be treated except in special situations such as pregnancy or urological invasive procedures [2,3]. This condition has an extremely variable incidence according to several factors such as age, sex, and comorbidities, from 1–5% in healthy pre-menopausal females to 23–89% in patients with spinal cord injuries [2,3]. These findings make UTI and ABU an important field of interest for antimicrobial stewardship strategies to optimise antimicrobial prescription and to reduce inappropriate urine culture requests [4,5].

The European Committee on Antimicrobial Susceptibility Testing (EUCAST) modified in 2020 the traditional categories “S” (susceptible), “I” (intermediate) and “R” (resistant) into “susceptible, standard dose”, “susceptible, increased exposure” and “resistant”, respectively [6]. These changes were particularly important especially for *Pseudomonas aeruginosa* where previously multi-susceptible isolates were described from that moment as “I” for first-line antibiotics such as cefepime, ceftazidime, and piperacillin/tazobactam; on the other hand, these modifications did not affect antibiogram interpretations for meropenem [6]. As a consequence, overprescription of carbapenems was described in the treatment of infections due to “wild type” (wt) *P. aeruginosa* [7]. In this context, we recently highlighted how the use of carbapenems for the treatment of bloodstream infections due to wt *P. aeruginosa* did not improve the outcome of these patients [8].

The aim of the present study was to evaluate (i) the impact of EUCAST 2020 definitions on antimicrobial prescription in UTI/ABU due to *P. aeruginosa*, (ii) the amount of inappropriately treated ABU and the related characteristics, and (iii) the role of infectious disease specialists in this context.

## 2. Materials and Methods

This was an observational, retrospective study. We enrolled all the patients with *P. aeruginosa* isolation from urine culture that were admitted to our tertiary care hospital from January 2018 to December 2021. Exclusion criteria were as follows: (i) age < 18 years; (ii) admission to the intensive care unit; (iii) exitus within 48 h from admission; (iv) pregnancy; (v) urine culture requested for invasive urological procedures; (vi) patients who did not provide their consent for retrospective observational studies.

Demographic, clinical, and microbiological data were collected on an ad hoc electronic case report form.

Characteristics of study population, cases of UTIs and ABUs were evaluated and compared between period A (2018–2019, before EUCAST 2020 update) and period B (2020–2021, after EUCAST 2020 update) such as antibiotic prescription, *P. aeruginosa* susceptibility pattern. In addition to this, the role of the infectious disease specialist was evaluated in terms of antimicrobial prescription as a consultant. Moreover, for those cases in which urine culture resulted positive for wt *P. aeruginosa*, we analysed antibiotic treatments, comparing anti-*Pseudomonas* cephalosporins—defined as cepefime and ceftazidime—and piperacillin/tazobactam versus carbapenems, ceftolozane/tazobactam, and ceftazidime/avibactam prescriptions.

UTIs and ABUs were defined according to the 2024 guidelines of the European Association of Urology (EAU) and of the Infectious Diseases Society of America. In particular ABUs were defined as the isolation of bacteria (>100,000 CFU/mL) from urine without any clinical signs of infection or symptoms; the occurrence of symptoms and signs (such as fever, flank or pelvic pain, frequency or urgency, dysuria) in the same context defined a UTI [3,9,10]. We defined those strains with an antibiogram interpretation of “I” and “R” in the period A and with an antibiogram interpretation of “R” in the period B as “non susceptible” to a certain antibiotic.

Statistical analysis was performed using SPSS version 23. In particular, parametric (Student T-test) and non-parametric (Mann–Whitney U-test with or without Yates correction as appropriate) tests were performed for the evaluation of continuous and non-continuous variables according to their distribution, as appropriate. Odds ratios with 95% confidence intervals were calculated for univariate and multivariate analysis with logistic regression.

The study was conducted consistently with the good clinical practice guidelines and the Declaration of Helsinki. Patients provided a written informed consent for the participation to retrospective observational studies. Ethical approval was waived according to the local ethical committee in relation to the retrospective observational design of the study.

## 3. Results

**Figure 1:**
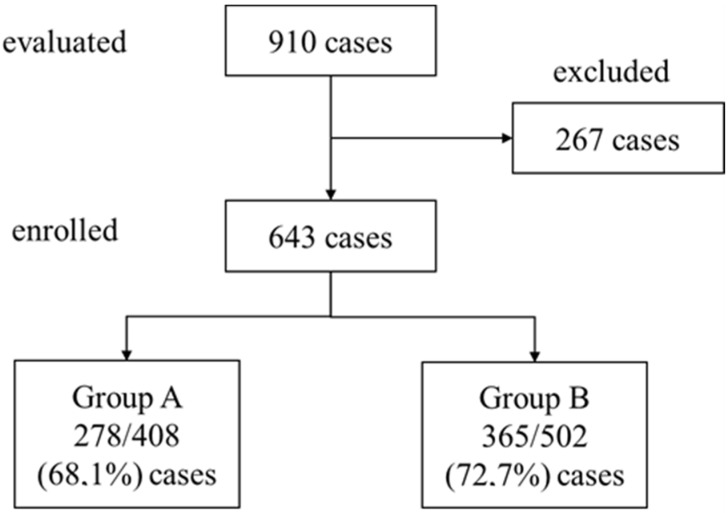
Study enrolment flow chart.

We evaluated 910 patients (408 in period A and 502 in period B) with a urine culture positive for *P. aeruginosa*, admitted to our hospital from January 2018 to December 2021; 267 cases were excluded according to inclusion and exclusion criteria so that the whole study population consisted of 643 cases, 278/408 (68.1%) in period A and 365/502 (72.7%) in period B (Figure 1).

### 3.1. Asymptomatic Bacteriuria

In 418 cases out of 643 (65%), the positive urine culture was associated with an ABU, 169/278 (60.8%) and 249/365 (68.2%) in period A and B, respectively (*p* = 0.0504). Table 1 described the characteristics of cases of ABUs in the whole study population and in period A and period B. In total, 153 out of 418 (36.6%) cases of bacteriuria underwent an antibiotic treatment during the study period, 70/169 (41.4%) in period A and 83/249 (33.3%) in period B (*p* = 0.092).

Carbapenems were administered in 49/153 (32%) cases, 15/70 (21.4%) in period A and 34/83 (41%) in period B with a statistically significant difference (*p* = 0.016); anti-*Pseudomonas* cephalosporins were administered in 14/153 (9.2%) cases, 11/70 (15.7%) cases in period A and in 3/83 (3.6%) cases in period B (*p* = 0.021). Piperacillin/tazobactam, ciprofloxacin, ceftazidime/avibactam, and ceftolozane/tazobactam were administered in 14/70 (20%), 21/70 (30%), 1/70 (1.4%) and 3/70 (4.3%) in period A and 27/83 (32.5%), 16/83 (19.3%), 4/83 (4.8%) and 0 cases in period B, respectively (*p* > 0.1, *p* > 0.1, *p* > 0.1 and *p* > 0.1).

Treatment duration was <7 days in 12/70 (15.9%) and 16/83 (19.3%), between 7 and 14 days in 51/70 (72.9%) and 55/83 (66.3%), >14 days in 7/70 (10%) and 12/83 (14.5%) in period A and period B, respectively (*p* > 0.1).

We also compared patients with ABU in which at least one antibiotic was administered (153/418, 36.6%) with patients with ABU without antibiotic treatment (265/418, 63.4%) (Table 2). These two groups were homogeneous for sex, age, comorbidities (evaluated by Charlson comorbidity index), urinary catheterisation, and multiple bacterial isolates in urine culture. The antimicrobial susceptibility patterns of *Pseudomonas* isolates were also similar between the two groups (treated vs. untreated ABUs). In particular, carbapenem non-susceptible (NS) isolates were detected in 13/153 (8.5%) and 35/265 (13.2%) cases (*p* > 0.1), anti-*Pseudomonas* cephalosporins NS isolates in 27/153 (17.6%) and 50/265 (18.9%) cases (*p* > 0.1) and piperacillin/tazobactam NS isolates in 46/153 (30.1%) and 87/265 (32.8%) cases in treated and untreated ABU patients, respectively.

### 3.2. Urinary Tract Infections (UTIs)

Table 3 describes cases of UTI in the study population (225/643, 35%), period A (109/278, 39.2%), and period B (116/365, 31.8%) (*p* = 0.0504). The groups were comparable for gender, age, and Charlson comorbidity index. Moreover, permanent urinary catheters were detected in 93/109 (85.3%) and 89/116 (76.7%) cases in period A and period B, respectively (*p* > 0.1) while a polymicrobial urinary isolate was characterised in 61/109 (56%) cases in period A and 54/116 (46.6%) cases in period B (*p* > 0.1). Low tract UTIs were diagnosed in 22/109 (20.1%) and 20/116 (17.2%) of cases in period A and period B, respectively (*p* > 0.1). In 8/62 (12.9%) of cases in period A and in 8/76 (10.5%) in period B concomitant positive blood cultures were observed (*p* > 0.1). Antibiotic susceptibility patterns were similar for isolated in both period in terms of resistance to carbapenems, anti-*Pseudomonas* cephalosporins, piperacillin/tazobactam, ceftolozane/tazobactam, ceftazidime/avibactam, and ciprofloxacin (*p* > 0.1).

Finally, also outcome was comparable between the two periods (positive clinical response after 5–7 days 82.6% vs. 88.8% [*p* > 0.1], in-hospital all-cause mortality 11% vs. 14.6% [*p* > 0.1], in-hospital UTI-related mortality 0.9% vs. 2.6% [*p* > 0.1] in period A vs. period B, respectively).

According to the aims of the present study, in order to evaluate the impact of new EUCAST definitions, we analysed the treatment of UTI due to wt *P. aeruginosa* (cases with isolation of a strain with resistance to anti-*Pseudomonas* cephalosporins, piperacillin/tazobactam, and ciprofloxacin were excluded from this analysis). As a consequence, 189/225 (84%) cases were evaluated, 90/109 (82.6%) in period A and 99/116 (85.3%) in period B. Table 4 describes our findings. The cases analysed in the two periods were comparable for gender, age, and comorbidities (Charlson comorbidity index). UTIs were related to a permanent urinary catheter in 76/90 (84.4%) and 74/99 (74.7%) cases in period A and period B (*p* > 0.1); in period A, a polymicrobial isolate was detected in 54/90 (60%) cases, while in period B it was detected in 42/99 (42.4%) with *p* > 0.1. Diagnosis of low urinary tract infection was established in 19/90 (21.1%) and 18/99 (18.2%) of cases in period A and period B, respectively (*p* > 0.1). Blood cultures resulted concomitantly positive in 7/48 (14.6%) cases in period A and in 6/65 (9.2%) cases in period B (*p* > 0.1).

Piperacillin/tazobactam and anti-*Pseudomonas* cephalosporins were administered in 17/87 (19.5%) and 18/87 (20.7%) cases in period A and 30/85 (35.3%) and 5/85 (5.9%) cases in period B (*p* = 0.032 and *p* = 0.009, respectively); use of carbapenems was similar in the two periods (34.5% vs. 42.4%, *p* > 0.1). Outcome analysis showed no differences in the two periods (positive clinical response after 5–7 days 85.6% vs. 89.9% [*p* > 0.1], in-hospital all-cause mortality 7.8% vs. 13.1% [*p* > 0.1], in-hospital UTI-related mortality 0% vs. 3% [*p* > 0.1] in period A vs. period B, respectively).

### 3.3. Use of Antibiotics in ABUs and UTIs

In order to evaluate the overall impact of EUCAST new definitions in the setting of positive urine culture, we analyse all the cases in which at least an antibiotic was prescribed and administered in both ABUs and UTIs due to wt *P. aeruginosa* (cases with isolates resistant to anti-*Pseudomonas* cephalosporins and/or piperacillin/tazobactam were excluded from this analysis). Table 5 described our findings. Data were available for 303 cases, 151 in period A and 152 in period B. Piperacillin/tazobactam were prescribed in 30/151 (19.9%) cases in period A and 52/152 (34.2%) cases in period B (*p* = 0.005) while anti-*Pseudomonas* cephalosporins in 28/151 (18.5%) cases in period A and 9/152 (5.9%) cases in period B (*p* = 0.001). Administration of carbapenems was reported in 42/151 (27.8%) and 63/152 (41.4%) cases (*p* = 0.013), ciprofloxacin in 37/151 (24.5%) and 24/152 (15.8%) cases (*p* = 0.059), ceftazidime/avibactam in 3/151 (2%) and 13 (8.6%) cases (*p* = 0.022) in period A and period B, respectively.

Table 6 compared treated cases (UTI and ABU) to untreated cases (UTI and ABU) in order to evaluate the possible elements that guide clinicians in treatment decision. Treated cases (group A) were more frequent in females (176/363, 48.5% vs. 164/280, 58.6% in group B, *p* = 0.011). Age, comorbidities evaluated by Charlson comorbidity index, urinary catheter-related infections, and polymicrobial isolations from urine culture were similar in treated and untreated cases. In group A 30/363 (8.3%) cases were due to carbapenem-NS *P. aeruginosa* isolates, while in group B they are 40/280 (14.3%) (*p* = 0.015). Incidence of isolates non susceptible to anti-*Pseudomonas* cephalosporins, piperacillin/tazobactam, ciprofloxacin, ceftolozane/tazobactam, and ceftazidime/avibactam were similar in group A e group B (*p* > 0.1). Positive blood cultures were detected in 16/176 (9.1%) cases in group A and 0/113 (0%) in group B (*p* < 0.001).

### 3.4. Infectious Diseases Consultation

An infectious disease consultation was requested in 131/418 (31.3%) cases of ABUs in the study period, 44/183 (28.6%) in treated ABUs and 87/265 (32.8) in non-treated cases (*p* > 0.1); in 94/131 (71.8%) the infectious diseases consultant did not suggest antibiotic treatment or stopped the on-going one. In particular, this kind of consultation was reported in 27/46 (58.7%) and 67/85 (78.8%) cases in period A and period B, respectively (*p* = 0.025).

## 4. Discussion

In the setting of antimicrobial stewardship programmes, complicated UTI (cUTI) and ABU represent a challenging field of action. Antibiotic overprescription is quite common in these cases; inappropriate antimicrobial use was described in 32–93% of such cases in older adults [11,12]. This antibiotic misuse is related to the development of antimicrobial resistance, adverse events, increasing incidence of *Clostridiodes difficile* infection [9]. As recently described, 2020 EUCAST updates could be linked to carbapenem overprescription in infections due to *P. aeruginosa* without any improvement in terms of outcome, even in patients with bloodstream infection [8]. We aimed to describe antibiotic use in cUTI and ABU due to *P. aeruginosa* and the impact on prescription of 2020 EUCAST new indications on antimicrobial susceptibility testing interpretation.

Our findings showed a relatively high amount of ABUs (65% of the whole study population) with a tendency to increase in the second period of observation that nearly achieved the threshold of statistical significance (*p* = 0.0504). These data are consistent with the literature. ABUs was reported to be up to 50% of all urine cultures in older adult residents in nursing homes, but this percentage increased for spinal injured patients and in the presence of an indwelling urinary catheter [11,13]. According to Van Buul and colleagues, inappropriate antimicrobial prescriptions in “presumed UTIs” were extremely common, from 32% to 93% of cases [12]. We found a total of 153 cases (36.6%) of treated ABUs, positioning our hospital in the lower part of the range. Moreover, the percentage of treated ABUs decreased from 41.4% to 33.3% from period A to period B without reaching statically confirmed significance but with a positive trend. This improvement was also confirmed by the increased number and impact of infectious diseases specialists’ consultation in this setting. Consultations with indication to discontinue the ongoing therapy or not to start at all an antibiotic treatment significantly rase from 58.7% in period A to 78.8% in period B (*p* = 0.025). The positive impact of consultation on patients’ outcome, even in severe cases such as Gram-negative bloodstream infections, is quite well described in the literature [14]. In the UTI setting, most studies were focused on treatment duration and training intervention [10,15].

Concerns arise from data about carbapenems’ prescription in ABUs. Obviously, all the prescription in this setting represented a misuse. However, the significantly increased rate of administration of carbapenems could be partially explained by the introduction of EUCAST 2020 new antibiogram interpretation definitions as we have previously shown in the setting of bloodstream infections due to *P. aeruginosa* [8].

As described in Table 2, we did not detect any significant risk factors for antibiotic prescription in ABU. A recently published paper, identified as factors related to the inappropriate treatment of ABU, misinterpreted signs and symptoms such as abdominal pain, falls, decreased urine output, urine characteristics and laboratory findings, and abnormal vital signs [16]. Although pyuria still represents one of the main elements to distinguish ABU from UTI, a very interesting trial published by Bilsen and colleagues in 2023 demonstrated how the currently available cut-off of pyuria are too low and can promote a UTI misdiagnosis with the related inappropriate antibiotic treatment in older women [17].

Analysing UTI cases, we detected a comparable situation. The characteristics of the patients were similar in the two periods of observation. The analysis of the treatments showed a significant reduction of anti-*Pseudomonas* cephalosporins prescriptions while the administration of piperacillin/tazobactam significantly increased (Table 4). Cases with isolation of isolates with resistance to piperacillin/tazobactam and/or anti-*Pseudomonas* cephalosporins were excluded from this analysis. More interestingly, we analysed antibiotic prescription in the whole “treated population”, such as cases of UTI plus cases of treated ABU (Table 5). As well-known and previously specified, ABUs have no indication for treatment. Nevertheless, we decided to evaluate the use of antibiotics also in this setting because the main objective of the study was to understand the impact of the 2020 EUCAST new definitions on the “prescription behaviour” that involved both correct and incorrect prescriptions. Moreover, we have to consider that cases retrospectively defined as ABUs, in medias res were incorrectly considered as real infection by the prescribing physician (this is the reason why antibiotics were prescribed in these cases). Our data confirmed the impact of the EUCAST 2020 new definitions on the antibiotic choice as previously reported also by Munting and colleagues [7]. Carbapenems and ceftazidime/avibactam prescription rate significantly increased while anti-*Pseudomonas* cephalosporins dramatically decreased. Surprisingly, both in this setting and in UTIs described in Table 4, piperacillin/tazobactam was significantly more administered in period B. Probably the well-known excellent urinary concentration of the drug and the relatively low rate of adverse events could have a role in this setting. Moreover, these suggestions were confirmed by recent studies that showed no differences in terms of clinical cure between piperacillin/tazobactam and carbapenems for UTI due to extended spectrum β-lactamases producing Gram-negative microorganisms [18].

To our opinion, these findings confirmed the field of urinary infection as an important target of antimicrobial stewardship programmes. Considering the elevated number of ABU, the first step of such a programme should be in the diagnostic setting. Interventions at different levels could be implemented: tools for clinicians to discourage urine culture and urinalysis without a specific indication and educational projects are only a few examples [19]. Winkler and colleagues showed a reduction in urine culture requests from 97% to 23% in patients prior to coronary artery bypass grafting after the removal of such exams from the preoperative set without any significant increase in post-operative urinary tract infections [20]. Another interesting approach was described by Langford and colleagues [11]. These authors elaborated a score, called “BLADDER score” whose application allowed antibiotic use reduction from 40.55 DDD per 1000 patients days to 25.96 DDD per 1000 patients days. This score evaluated presence of blood in urine, incontinence, abdominal or suprapubic pain, dysuria, fever, and increased frequency of urination.

As mentioned above, an alarming concern arose about the increase in carbapenems’ prescription in cases with the isolation of a strain susceptible to piperacillin/tazobactam and anti-*Pseudomonas* cephalosporins. An interesting intervention in this setting was proposed by Munting and colleagues [21]: masking meropenem in antibiogram, these authors obtained a reduction in meropenem prescription from 25.3% before the intervention to 8.4% after the intervention.

Finally, other specific interventions could be performed in the post-analytical phase. Gohil and colleagues recently published an interesting cluster randomised trial where a computerised composite stewardship intervention (education, feedback, risk for MRd organisms’ isolation) was compared to “routine stewardship”. In the intervention group, the prescription of extended spectrum antibiotics was reduced by 17.4% [22].

The present study has several limitations such as its retrospective observational design, the monocentric nature, and the focus limited only to a specific pathogen. On the other hand, these findings could be of interest for stewardship programme implementation both at diagnostic and therapeutic levels.

## 5. Conclusions

In conclusion, we described an increase in carbapenems’ prescription after the 2020 EUCAST update about the interpretation of the *P. aeruginosa* susceptibility pattern with a concomitant significant reduction in anti-*Pseudomonas* cephalosporins’ use in a setting with a high percentage of ABU. Infectious diseases specialists’ consultations showed an increased role in avoiding antimicrobial inappropriate prescriptions. These findings could represent the basis of a wide antimicrobial and diagnostic stewardship programme.

## Figures and Tables

**Table 1:** Cases of asymptomatic bacteriuria: period A vs. period B.

	Study Population (*n* = 643)	Period A (*n* = 278)	Period B (*n* = 365)	*p*
Asymptomatic bacteriuria cases	418 (65%)	169 (60.8%)	249 (68.2%)	0.0504
Asymptomatic bacteriuria cases with antibiotic administration	153/418 (36.6%)	70/169 (41.4%)	83/249 (33.3%)	0.092
Antibiotic treatment				
- carbapenem	49 (32%)	15 (21.4%)	34 (41%)	**0.016**
- antipseudomonal cephalosporins	14 (9.2%)	11 (15.7%)	3 (3.6%)	**0.021**
- piperacillin/tazobactam	41 (26.8%)	14 (20%)	27 (32.5%)	>0.1
- ciprofloxacin	37 (24.2%)	21 (30%)	16 (19.3%)	>0.1
- ceftazidime/avibactam	5 (3.3%)	1 (1.4%)	4 (4.8%)	>0.1
- ceftolozan/tazobacta	3 (2%)	3 (4.3%)	0	>0.1
others	16 (10.5%)	6 (8.6%)	10 (12%)	>0.1
Duration of treatment				
- <7 days	28 (18.3%)	12 (15.9%)	16 (19.3%)	>0.1
- 7–14 days	106 (69.3%)	51 (72.9%)	55 (66.3%)	
- >14 days	19 (12.4%)	7 (10%)	12 (14.5%)	

**Table 2:** Cases of asymptomatic bacteriuria: characteristics of treated and not treated cases.

	Asymptomatic Bacteriuria (*n* = 418)	With Antibiotic Treatment (*n* = 153, 36.6%)	Without Antibiotic Treatment (*n* = 265, 63.4%)	*p*
Females	242 (57.9%)	83 (54.2%)	159 (60%)	>0.1
Age, median (IQR)	83 (74–88)	83 (76–89)	83 (73–88)	>0.1
Charlson comorbidity index	7 (5–8)	6.5 (5–8)	7 (5–8)	>0.1
Urinary catheter	346 (82.8%)	122 (79.7%)	224 (84.5%)	>0.1
Multiple bacterial isolates from urine culture	247 (59.1%)	91 (59.5%)	156 (58.9%)	>0.1
*P. aeruginosa* antibiogram:				
- carbapenem-NS	48 (11.5%)	13 (8.5%)	35 (13.2%)	>0.1
- antipseudomonal cephalosporins-NS	77 (18.4%)	27 (17.6%)	50 (18.9%)	>0.1
- ceftolozane/tazobactam-NS	7/224 (3.1%)	3/79 (3.8%)	4/145 (2.8%)	>0.1
- ceftazidime/avibactam-NS	12/223 (5.4%)	2/78 (2.6%)	10/145 (6.9%)	>0.1
- ciprofloxacin-NS	144 (34.4%)	53 (34.6%)	91 (34.3%)	>0.1
- piperacillin/tazobactam-NS	133 (31.8%)	46 (30.1%)	87 (32.8%)	>0.1
Positive blood culture	0/151	0/47	0/104	>0.1

**Table 3:** Cases of urinary tract infections: period A vs. period B.

	Study Population (225/643, 35%)	Period A (109/278, 39.2%)	Period B (116/365, 31.8%)	*p* 0.0504
Female sex	98 (43.6%)	46 (42.2%)	52 (44.8%)	>0.1
Age, median (IQR)	82 (73–86)	82 (74.75–86)	81 (72–86)	>0.1
Charlson comorbidity index	6 (5–8)	6 (5–8)	6 (5–8)	>0.1
Urinary catheter	182 (80.9%)	93 (85.3%)	89 (76.7%)	>0.1
Multiple bacterial isolates from urine culture	115 (51.1%)	61 (56%)	54 (46.6%)	>0.1
Low tract UTIs	42 (18.7%)	22 (20.1%)	20 (17.2%)	>0.1
*P. aeruginosa* antibiogram:				
- carbapenem-NS	22 (9.8%)	13 (11.9%)	9 (7.8%)	>0.1
- antipseudomonal cephalosporins-NS	38 (16.9%)	20 (18.3%)	18 (15.5%)	>0.1
- ceftolozane/tazobactam-NS	6/110 (5.5%)	1/8 (12.5%)	5/102 (4.9%)	>0.1
- ceftazidime/avibactam- NS	8/108 (7.4%)	1/6 (16.7%)	7/102 (6.9%)	>0.1
- ciprofloxacin-NS	78 (34.7%)	44 (40.4%)	34 (29.3%)	0.082
- piperacillin/tazobactam-NS	63 (28%)	33 (30.3%)	30 (25.9%)	>0.1
Concomitant positive blood culture	16/138 (11.6%)	8/62 (12.9%)	8/76 (10.5%)	>0.1
Positive clinical response after 5/7 days	193 (85.8%)	90 (82.6%)	103 (88.8%)	>0.1
In-hospital all-cause mortality	29 (12.9%)	12 (11%)	17 (14.6%)	>0.1
In-hospital UTI-related mortality	4 (1.8%)	1 (0.9%)	3 (2.6%)	>0.1

**Table 4:** Cases of urinary tract infections: clinical characteristics and antibiotic treatment. Cases with resistance to piperacilline/tazobactam and anti-pseudomonal cephalosporins were excluded.

	Study Population (189/225, 84%)	Period A (90/109, 82.6%)	Period B (99/116, 85.3%)	*p*
Females	84 (44.4)	39 (43.3)	45 (45.5)	>0.1
Age, *median (IQR)*	81.5 (73–86)	82 (74–86)	81 (71.5–86)	>0.1
Charlson comorbidity index	6 (5–8)	6 (5–8)	6 (5–8)	>0.1
Admission to Infectious Diseases ward	10 (5.3)	3 (3.3)	7 (7.1)	>0.1
Urinary catheter	150 (79.4)	76 (84.4)	74 (74.7)	>0.1
Multiple bacterial isolates from urine culture	96 (50.8)	54 (60)	42 (42.4)	**0.016**
Low tract UTI	37 (19.6)	19 (21.1)	18 (18.2)	>0.1
Positive blood cultures	13/113 (11.5)	7/48 (14.6)	6/65 (9.2)	>0.1
Antibiotic treatment:	*n* = 172	*n* = 87	*n* = 85	
- piperacillin/tazobactam	47 (27.3)	17 (19.5)	30 (35.3)	**0.032**
- anti-pseudomonal cephalosporins	23 (13.4)	18 (20.7)	5 (5.9)	**0.009**
- carbapenems	66 (38.4)	30 (34.5)	36 (42.4)	>0.1
- ciprofloxacin	25 (14.5)	16 (18.4)	9 (10.6)	>0.1
other	39 (22.7)	15 (17.2)	24 (28.2)	>0.1
Duration of treatment				
- <7 days	28/177 (15.8)	18/85 (21.2)	10/92 (10.9)	0.083
- 7–14 days	126/177 (71.2)	54/85 (63.5)	72/92 (78.3)	
- >14 days	23/177 (13)	13/85 (15.3)	10/92 (10.9)	
Positive clinical response after 5/7 days	166 (87.8)	77 (85.6)	89 (89.9)	>0.1
In-hospital all-cause mortality	20 (10.6)	7 (7.8)	13 (13.1)	>0.1
In-hospital UTI-related mortality	3 (1.6)	0	3 (3)	>0.1

**Table 5:** Antibiotic administered in urinary tract infections and treated asymptomatic bacteriurias; comparison between period A and period B.

	Study Population *n* = 310	Period A (*n* = 151)	Period B (*n* = 159)	*p*
Antibiotic therapies:	*n* = 303	*n* = 151	*n* = 152	
- piperacillin/tazobactam	82 (27.1)	30 (19.9)	52 (34.2)	**0.005**
- anti-pseudomonal cephalosporins	37 (12.2)	28 (18.5)	9 (5.9)	**0.001**
- carbapenems	105 (34.7)	42 (27.8)	63 (41.4)	**0.013**
- ciprofloxacin	61 (20.1)	37 (24.5)	24 (15.8)	0.059
- ceftolozane/tazobactam	13 (4.3)	7 (4.6)	6 (3.9)	>0.1
- ceftazidime/avibactam	16 (5.3)	3 (2)	13 (8.6)	**0.022**
other	26 (8.6)	16 (10.6)	10 (6.6)	>0.1

**Table 6:** Characteristics of treated and untreated cases.

	Study Population (*n* = 643)	Group A (Treated 363/643, 56.5%)	Group B (Untreated 280/643, 43.5%)	*p*
Females	340 (52.9)	176 (48.5)	164 (58.6)	**0.011**
Age, *median* (*IQR*)	83 (74–88)	82 (73.25–87)	83 (74.75–88)	>0.1
Charlson comorbidity index	6 (5–8)	6 (5–8)	7 (5–8)	>0.1
Urinary catheter	528 (82.1)	292 (80.4)	236 (84.3)	>0.1
Multiple bacterial isolates from urine culture	281 (43.7)	166 (45.7)	115 (41.1)	>0.1
*P. aeruginosa* antibiogram:				
- carbapenem NS	70 (10.9)	30 (8.3)	40 (14.3)	**0.015**
- antipseudomonal cephalosporins NS	115 (17.9)	60 (16.5)	55 (19.6)	>0.1
- ceftolozane/tazobactam NS	13 (3.9)	9 (4.9)	4 (2.6)	>0.1
- ceftazidime/avibactam NS	20 (6)	10 (5.6)	10 (6.6)	>0.1
- ciprofloxacin NS	222 (34.5)	126 (34.7)	96 (34.3)	>0.1
- piperacillin/tazobactam NS	196 (30.5)	103 (28.4)	93 (33.2)	>0.1
Positive blood cultures	16/289 (5.5)	16/176 (9.1)	0/113 (0)	**<0.001**

## Data Availability

The datasets analysed during the current study are not publicly available due to privacy protection reasons but are available from the corresponding author on reasonable request.
